# Association between frailty index and all-cause mortality in hospitalized elderly patients with osteoporotic fractures: a retrospective cohort study

**DOI:** 10.3389/fmed.2025.1697650

**Published:** 2025-11-21

**Authors:** Peng Zhou, Ke Lu, Ya-qin Gong, Jian Jin, Wen-bin Hu, Chong Li, Yi Yin

**Affiliations:** 1 Department of Orthopedics, Gusu School, Nanjing Medical University, The First People's Hospital of Kunshan, Suzhou, Jiangsu, China; 2 Kunshan Biomedical Big Data Innovation Application Laboratory, Suzhou, Jiangsu, China; 3 Information Department, Affiliated Kunshan Hospital of Jiangsu University, Suzhou, Jiangsu, China; 4 Kunshan Municipal Health and Family Planning Information Center, Suzhou, Jiangsu, China; 5 Chronic Disease Department, Kunshan Center for Disease Control and Prevention, Suzhou, Jiangsu, China

**Keywords:** frailty index, all-cause mortality, osteoporotic fractures, elderly patients, hospitalization

## Abstract

**Background:**

Osteoporotic fractures (OPF) represent a significant health concern among the elderly population. Frailty, a prevalent condition in this demographic, can be evaluated via the Frailty Index (FI). This study investigated the association between FI and all-cause mortality (ACM) in aged individuals with osteoporosis (OP).

**Methods:**

This retrospective cohort study included 19,332 patients who underwent surgical treatment for fractures at Kunshan First People’s Hospital between January 1, 2017, and August 31, 2023. Among these, 4,782 patients aged ≥ 50 years were diagnosed with OPF. The FI was developed based on 30 health indicators, and it requires the availability of at least 75% of the variables for all patients. Moreover, ACM was monitored from the time of hospitalization until death or the end of the study period. Data on the correlation between FI and ACM were statistically evaluated, including the Cox proportional hazard regression model, interaction test, smooth curve fitting, K-M survival curve, threshold effect, subgroup, and sensitivity analyses.

**Results:**

Among the 3,833 patients, the mean age was 68.77 years, with an average FI of 0.07. A substantial positive correlation was observed between FI and ACM (HR = 1.05, 95% CI: 1.03–1.07, *p* < 0.01). Importantly, a 0.033 increase in the FI score (equivalent to ~ 1 additional cumulative deficit) was related to a 17% higher risk of ACM (HR: 1.17, 95% CI: 1.10 to 1.24). Subgroup analyses further validated these findings across diverse demographic groups.

**Conclusion:**

This study establishes a significant correlation between the FI and ACM in elderly patients with OPF, underscoring the importance of frailty measurement in clinical management. These findings support the need for targeted interventions to improve outcomes in this high-risk population and emphasize the necessity of further research to develop effective screening and management strategies.

## Introduction

As the global population ages, the health challenges among elderly people have become a growing focus (1). Osteoporosis (OP) is a significant public health concern, affecting approximately 39.4% (2) of the elderly population in China. Globally, OPF occurs at an alarming rate of one every 3 s, amounting to approximately 8.9 million cases annually (3). These fractures remarkably impair patients’ quality of life (QoL) and are closely related to elevated mortality rates (4, 5). Studies highlight the high prevalence of OP among older people, particularly in women, with fracture incidence increasing substantially with advancing age (6, 7). The implications of OPF extend beyond reduced physical function to include prolonged hospitalization and long-term care requirements (3, 8). Among older people, OPF is frequently associated with factors such as decreased bone density and a heightened risk of falls (9, 10). Thus, early screening and timely treatment for OP are essential to reduce fracture risk and improve the QoL in this population (11, 12). As the current understanding of OP evolves, future research will focus on developing comprehensive intervention strategies to enhance skeletal health in elderly individuals.

The FI, a comprehensive tool for assessing frailty, incorporates multiple health indicators to evaluate an individual’s physiological reserves and resilience effectively (13–15). Studies have demonstrated a strong correlation between FI and health outcomes in the elderly, particularly in OPF patients (16–20). The presence of frail status may exacerbate post-fracture recovery challenges, increase the risk of complications, and affect overall mortality rates. Furthermore, frailty is not only related to fracture incidence but also closely associated with adverse outcomes such as postoperative recovery, duration of hospitalization, and readmission rates (21–25). Therefore, assessing frailty status in elderly patients is crucial for devising effective clinical interventions to improve their prognosis and QoL (26–30).

Studies have shown a significant correlation between FI and overall mortality rates (31, 32) for the elderly population. However, whether FI is uniformly associated with ACM in OF patients remains unclear. Investigating the role of FI in OPFs is crucial for improving clinical management and prognostic outcomes in elderly patients. This study not only advances the current understanding of the risk factors contributing to OP-related mortality but also serves as a valuable reference for guiding clinical practice. Therefore, this study aimed to investigate the association between the frailty index and all-cause mortality among elderly patients hospitalized with osteoporotic fractures.

## Materials and methods

### Ethical concern

This study was approved by the Ethics Committee of the Affiliated Kunshan Hospital of Jiangsu University (approval No. 2024-03-053-H00-K01), and conducted per the guidelines outlined in the Declaration of Helsinki. To maintain objectivity and unbiased investigation, all participants provided informed consent (in writing), and their identities were anonymized throughout the investigation.

### Study participants

**Figure 1:**
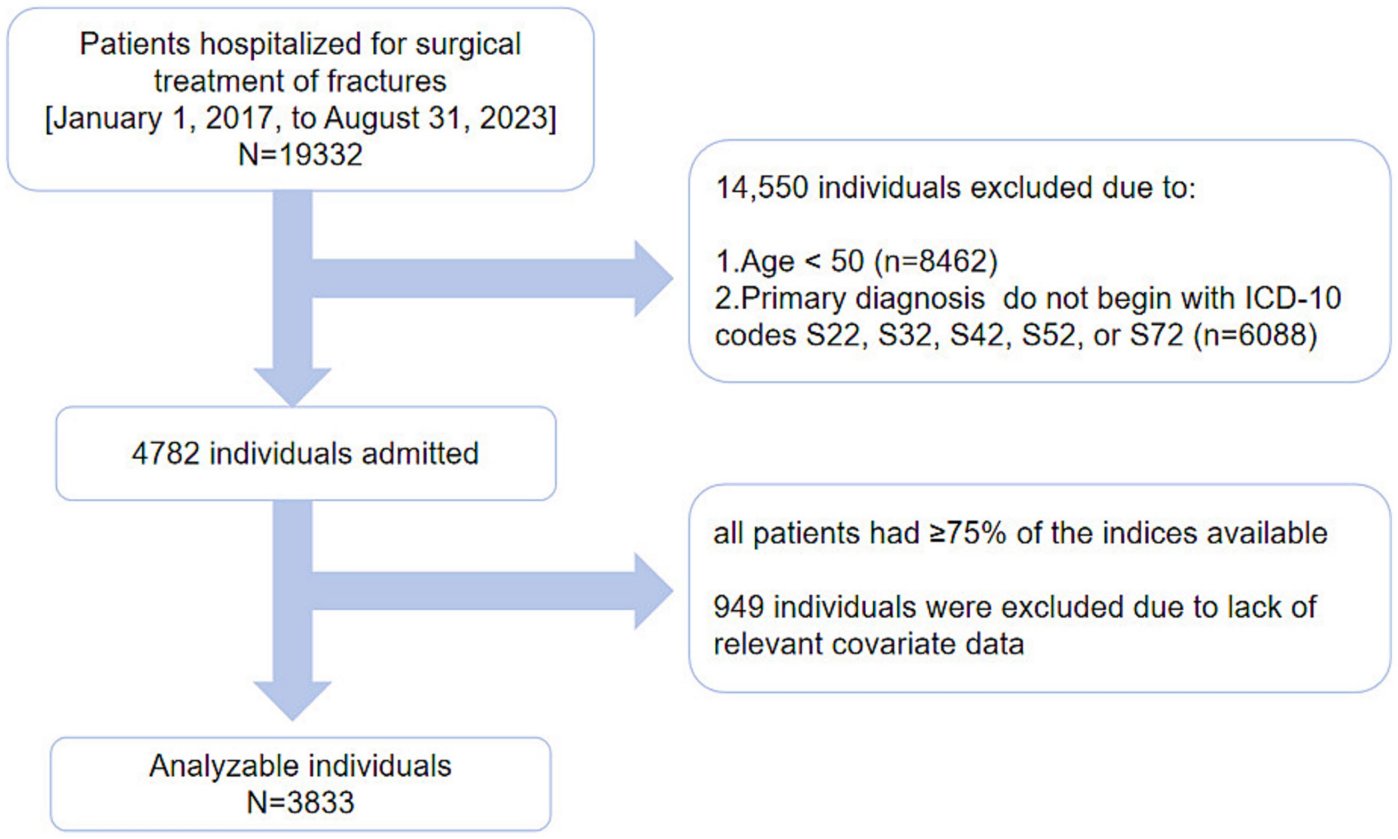
A conceptual diagram illustrating the study design.

We conducted a cohort study spanning from January 1, 2017, to August 31, 2023. It initially enrolled 19,332 patients who were treated for fractures at the First People’s Hospital of Kunshan. From this group, 8,462 patients under the age of 50 were excluded. A total of 4,782 patients with OPFs who underwent inpatient surgical treatment were included in the study. Patients with diagnoses corresponding to ICD-10 codes S22, S32, S42, S52, or S72, based on the International Statistical Classification of Diseases and Related Health Problems, 10th Revision, were selected. Blood tests were performed for all patients during their hospitalization. Blood samples (in fasting condition) were collected, and all clinical parameters were assessed within 3 days of admission. OP was diagnosed based on fractures and skeletal instability in the absence of bone metabolic disorders or a standard bone mineral density (BMD) T-score of −2.5 or lower, even in the absence of fractures (33). Data for ≥ 75% of the 30 indicators required for FI assessment, equivalent to at least 22 indicators (32), were available for all included patients. A total of 949 patients were excluded due to missing data on other covariates (As shown in Figure 1).

### Exposure and outcome variables

The construction of the FI was based on the methodology established by Galimberti et al. (32). It incorporated 30 variables that reflected the extent of pre-fracture comorbidities, pre-fracture medication use, and post-admission laboratory results. The final FI required the availability of at least 75% of these variables for each individual to maintain its comprehensive assessment. For each item, a score of “0” was assigned if deficits were absent, and a score of “1” indicated the presence of deficits. Abnormal laboratory values were determined based on reported laboratory reference ranges. The cumulative deficit count divided by the non-missing item numbers yielded a standardized score (ranging from 0 to 1, where 0 signifies no frailty and 1 represents complete frailty) (32).

Besides, ACM was defined as the time interval between the admission date for fracture-related hospitalization and the occurrence of death, transfer out of the area, or the study’s end date (April 24, 2024). We could not collect any relevant information about the cases transferred out of this region. All patients who were still alive before the end date of the study were included in the study. Mortality-related data for this study were obtained from the Jiangsu Provincial Death Registration System (PDRS).

### Covariate variables

Covariate variables, including age, body mass index (BMI), gender, smoking status, alcohol consumption, American Society of Anesthesiologists (ASA) score, and fracture type, were assessed. BMI was calculated by dividing weight (kg) by the square of height (m). Smoking was classified as current or former smoking within the preceding 12 months. Alcohol consumption was defined as drinking at least once per week over the past 12 months. The ASA score was employed to evaluate overall physical status. Fractures were categorized into the following types: thoracic vertebrae, lumbar vertebrae, wrist, proximal humerus, and femur (34). Some of these patients may have multiple fractures.

### Statistical analyses

Continuous data regarding patient demographics and clinical characteristics are depicted as mean ± standard deviation (SD) or median [1st quartile (Q1) to 3rd quartile (Q3)]. Normally distributed data were compared via independent two-tailed t-tests, while non-normally distributed data were analyzed using the Mann–Whitney U test. Categorical data are expressed as frequency (%), with variances evaluated via chi-squared tests. When the assumptions for the chi-squared test were not satisfied, the Fisher exact test was applied as an alternative. Cox proportional hazard regression models were used to assess the association between FI and ACM. The initial multivariate analysis comprised patient age, gender, BMI, alcohol consumption, smoking status, ASA score, and fracture type. A smooth curve fit was applied to generate a plot illustrating the correlation between FI and ACM. Moreover, Kaplan–Meier (K-M) survival curves were constructed to represent patient outcomes. Furthermore, subgroup analyses were conducted based on patient gender, age percentiles, BMI, smoking and drinking habits, ASA score (1, 2, ≥3), and fracture categories. The study also used likelihood ratio tests (LRT) to compare subgroup modifications and correlations. In the sensitivity analysis, follow-up times of 1, 2, 3, and 5 years were used, and K-M curves were generated for each time point to evaluate whether variations in follow-up duration influenced the relationship between FI and ACM in patients.

Data was statistically measured using R packages from The R Foundation^1^ and Empower Stats from X&Y Solutions.^2^ The significance threshold level was set at *p* < 0.05 for all two-tailed tests.

## Results

### Participant features

The current comprehensive data comprising ≥ 75% of the parameters (with at least 22 indicators/individual) were obtained for all patients. After excluding subjects with missing data (*n* = 949), approximately 3,833 patients were included in the final analysis (Figure 1).

Table 1 summarizes the process of constructing the FI. Table 2 presents the overall characteristics of the study cohort. The mean age of patients was 68.77 ± 11.26 years (interquartile range: 59.00–77.00 years). The participants were mostly females (*n* = 3,288, 67.71%), while males accounted for 32.29% (*n* = 1,544) of the sample. The mean BMI was 23.27 ± 3.29 kg/m^2^ (interquartile range: 20.93–25.35 kg/m^2^).

**Table 1:** Construction of the FI.

Deficit		Coding	Presence *n* (%)	Missing values *n* (%)
Cardiovascular disease				
1	Arrhythmia	No = 0; Yes = 1	63 (1.32)	–
2	Valvular disease	No = 0; Yes = 1	64 (1.34)	–
3	Peripheral Vascular disease	No = 0; Yes = 1	26 (0.54)	–
4	Hypertension	No = 0; Yes = 1	659 (13.78)	–
5	Ischemic disease	No = 0; Yes = 1	39 (0.82)	–
6	Thromboembolic	No = 0; Yes = 1	28 (0.59)	–
7	New York Heart Association (NYHA)	No = 0; Yes = 1	–	4,782 (100)
Other medical histories				
8	Endocrinological disorders	No = 0; Yes = 1	195 (4.08)	–
9	Neurologic illnesses	No = 0; Yes = 1	220 (4.60)	–
10	Hepatic disorders	No = 0; Yes = 1	20 (0.42)	–
11	Oncologic status	No = 0; Yes = 1	53 (1.11)	–
12	Psychiatric condition other than sleep disorders	No = 0; Yes = 1	19 (0.40)	–
13	Enteral disease	No = 0; Yes = 1	1 (0.02)	–
14	Gastric disease	No = 0; Yes = 1	6 (0.13)	–
15	Hematologic	No = 0; Yes = 1	0 (0.00)	–
16	Musculoskeletal disorder	No = 0; Yes = 1	4,782 (100)	–
17	Neuropathic pain	No = 0; Yes = 1	0 (0.00)	–
18	Pulmonary disease	No = 0; Yes = 1	96 (2.01)	–
19	Renal disease	No = 0; Yes = 1	36 (0.75)	–
Drugs consumption				
20	Anticoagulants (Anticoagulants prior to injury)	No = 0; Yes = 1	121 (2.53)	–
21	Antiplatelets (Antiplatelets prior to injury)	No = 0; Yes = 1	125 (2.61)	–
22	BetaBlocker (use of beta blocker prior to injury)	No = 0; Yes = 1	67 (1.40)	–
23	Sedative (use of sedatives in the past 3 months or more)	No = 0; Yes = 1	91 (1.90)	–
24	More than two other drugs (considering the number of drugs assumed before the admission in hospital)	≤2 drugs = 0; >2 drugs = 1	77 (1.61)	–
Laboratory measures				
25	Albumin first measure recorded (at baseline)	Defined as 1 if albumin level (g/dL) < 32	135 (2.82)	–
26	Total Bilirubin first measure recorded (at baseline)	Defined as 1 if total bilirubin level (umol/L) >20.2	–	4,782 (100)
27	Amylase first measure recorded (at baseline)	Defined as 1 if amylase level (UL) > 140	–	4,782 (100)
28	Creatinine first measure recorded (at baseline)	Defined as 1 if creatinine level (umol/L) < 53 or >106	959 (20.05)	60 (1.25)
29	SGPT first measure (at baseline) recorded	Defined as 1 if SGPT level (UL) < 10 or >50	444 (9.28)	61 (1.28)
30	SGOT first measure (at baseline) recorded	Defined as 1 if SGOT level (UL) < 8 or >33	880 (18.40)	61 (1.28)

FI, frailty index; NYHA, New York Heart Association; SGPT, Serum Glutamic Pyruvic Transaminase; SGOT, Serum Glutamic Oxaloacetic Transaminase.

**Table 2:** Characteristics of study participants.

Characteristics	Mean (SD) Median (Q1-Q3)^a^
Age, years	68.77 (11.26) 68.00 (59.00–77.00)
BMI, kg/m^2^	23.27 (3.29) 23.24 (20.93–25.35)
FI score, *N*	0.07 (0.04) 0.07 (0.04–0.07)

	*N* (%)
Sex, *N* (%)	
Female	3,288 (67.71%)
Male	1,544 (32.29%)
Smoke, *N* (%)	
No	4,228 (92.36%)
Yes	350 (7.65%)
Drink, *N* (%)	
No	4,341 (94.82%)
Yes	237 (5.18%)
ASA score, *N* (%)	
1	723 (12.38%)
2	3,120 (65.25%)
3	1,052 (22.00%)
4	18 (0.38%)
Fracture category, *N* (%)	
Thoracic vertebra	723 (15.12%)
Lumbar vertebra	1,290 (26.98%)
Wrist	410 (8.57%)
Proximal humerus	668 (13.97%)
Thighbone	1,691 (35.36%)
Death, *N* (%)	
No	4,205 (87.93%)
Yes	577 (12.07%)

a For continuous variables.

SD, standard deviation; Q1, first quartile; Q3, third quartile; CI, confidence interval; BMI, body mass index; FI, frailty index; ASA, American Society of Anesthesiologists.

The sample contained 350 smokers (7.65%) and 237 drinkers (5.18%). An ASA anesthesia score of 2 was the most prevalent category, with 3,120 individuals (65.25%). For ASA scores of 1, 3, and 4, the distribution was as follows: 723 individuals (12.38%), 1,052 individuals (22.00%), and 18 individuals (0.38%), respectively. In the thoracic vertebrae, lumbar vertebrae, wrist, proximal humerus, and thighbone, the corresponding numbers of fractures were 723 (15.12%), 1,290 (26.98%), 410 (8.57%), 668 (13.97%), and 1,691 (35.36%), respectively.

**Figure 2:**
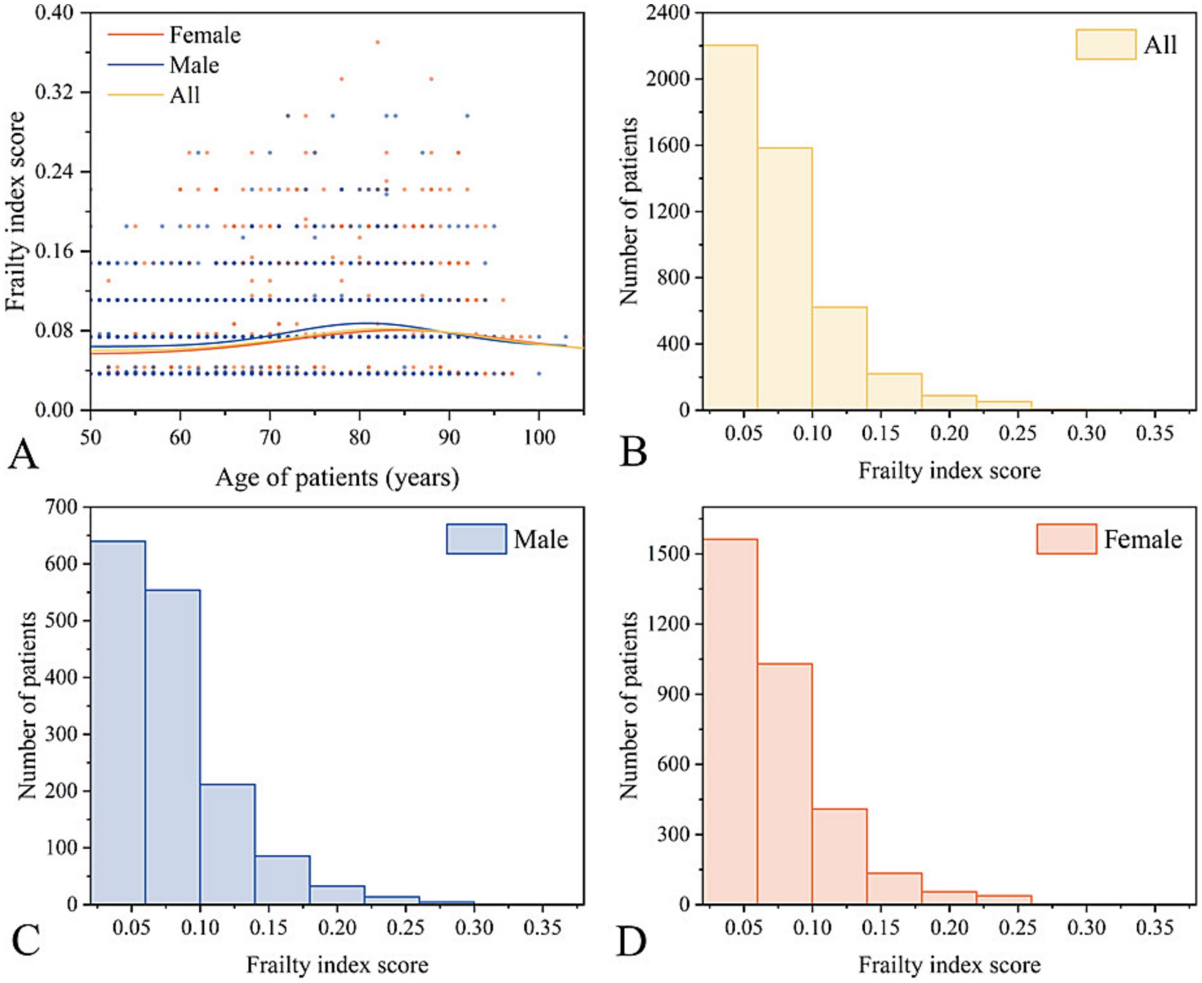
Overview of the frailty index. **(A,B)** General distribution of FI scores. Distribution of FI scores for males **(C)** and females **(D)**.

Figure 2 illustrates the distribution of FI values, with most values clustered < 0.10. The mean FI for all participants was 0.07 ± 0.04. The FI levels of male and female patients were similar, indicating a weak correlation between FI and age and an approximated overall mean level. A total of 577 patients (12.07%) had passed away by the end of the follow-up period.

### Correlation between FI and ACM

A total of 3 models were employed to examine the relationship between FI and ACM in OPFs (Table 3). In the unadjusted Model 1, a remarkable correlation between FI and ACM was observed (HR = 1.09, 95% CI: 1.08–1.11, *p* < 0.01). Model 2, which adjusted for age, gender, and BMI, demonstrated consistent findings, with FI remaining considerably correlated with ACM (HR = 1.05, 95% CI: 1.03–1.07, p < 0.01). In Model 3, after adjustments for smoking, alcohol consumption, ASA score, and fracture type, a persistent, substantial positive association between FI and ACM was found (HR = 1.05, 95% CI: 1.03–1.07, p < 0.01). (HR corresponds to the effect of each 0.01 increase in the FI score).

**Table 3:** Association between FI and ACM in different models.

	Model 1^a^ *N* = 4,782 HR (95%CI) *p*-value	Model 2^b^ *N* = 4,023 HR (95%CI) *p*-value	Model 3^c^ *N* = 3,833 HR (95%CI) *p*-value
FI^d^	1.09 (1.08, 1.11) <0.01	1.05 (1.03, 1.07) <0.01	1.05 (1.03, 1.07) <0.01

a No adjustment.

b Adjusted for sex, age, BMI.

c Adjusted for sex, age, BMI, smoke, drink, ASA, fracture category.

d HR refers to each 0.01 increase in the frailty index score.

FI, frailty index; ACM, all-cause mortality; HR, hazard ratio; BMI, body mass index; ASA, American Society of Anesthesiologists.

Overall, each 0.033-point increase in the FI score (approximately equivalent to one additional deficit) was related to a 17% higher risk of ACM (HR: 1.17, 95% CI: 1.10–1.24). This indicates that higher FI scores are associated with poorer prognoses for OPFs.

### Spline smoothing plot and threshold analysis

**Figure 3:**
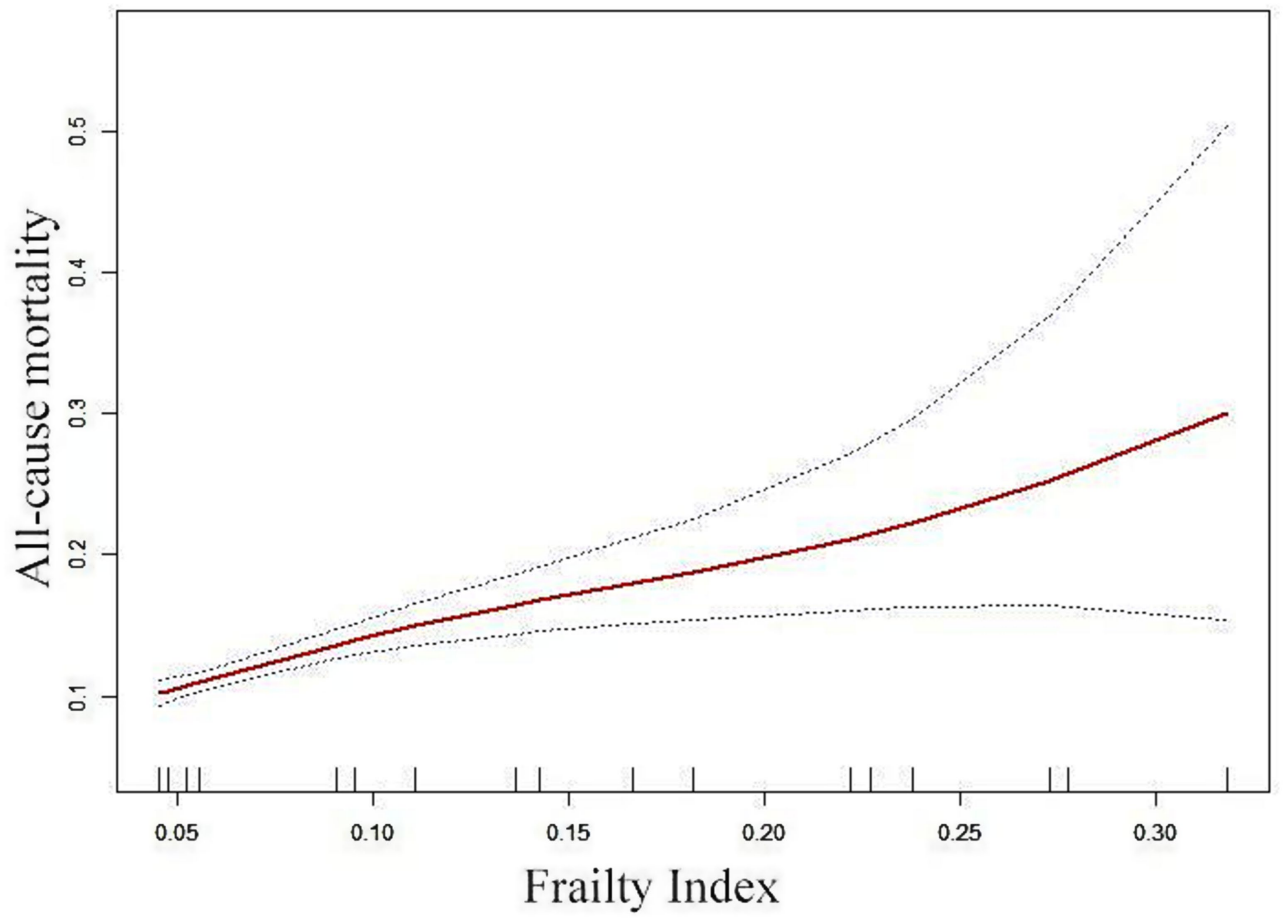
Adjusted smoothed curve analysis illustrates the FI and ACM correlation. The solid red line represents the fitted smooth curve, while the blue bands denote the 95% CI of the fit. The analysis was adjusted for gender, age, BMI, smoking status, alcohol consumption, ASA score, and fracture types.

The correlation between FI and ACM was assessed via graphical methods to examine its linearity (Figure 3). Generalized Additive Model (GAM) estimates demonstrated a distinct linear association between FI and ACM in the OPF population after adjusting for covariates. No inflection point was observed in the threshold effect analysis.

### K-M survival curve

**Figure 4:**
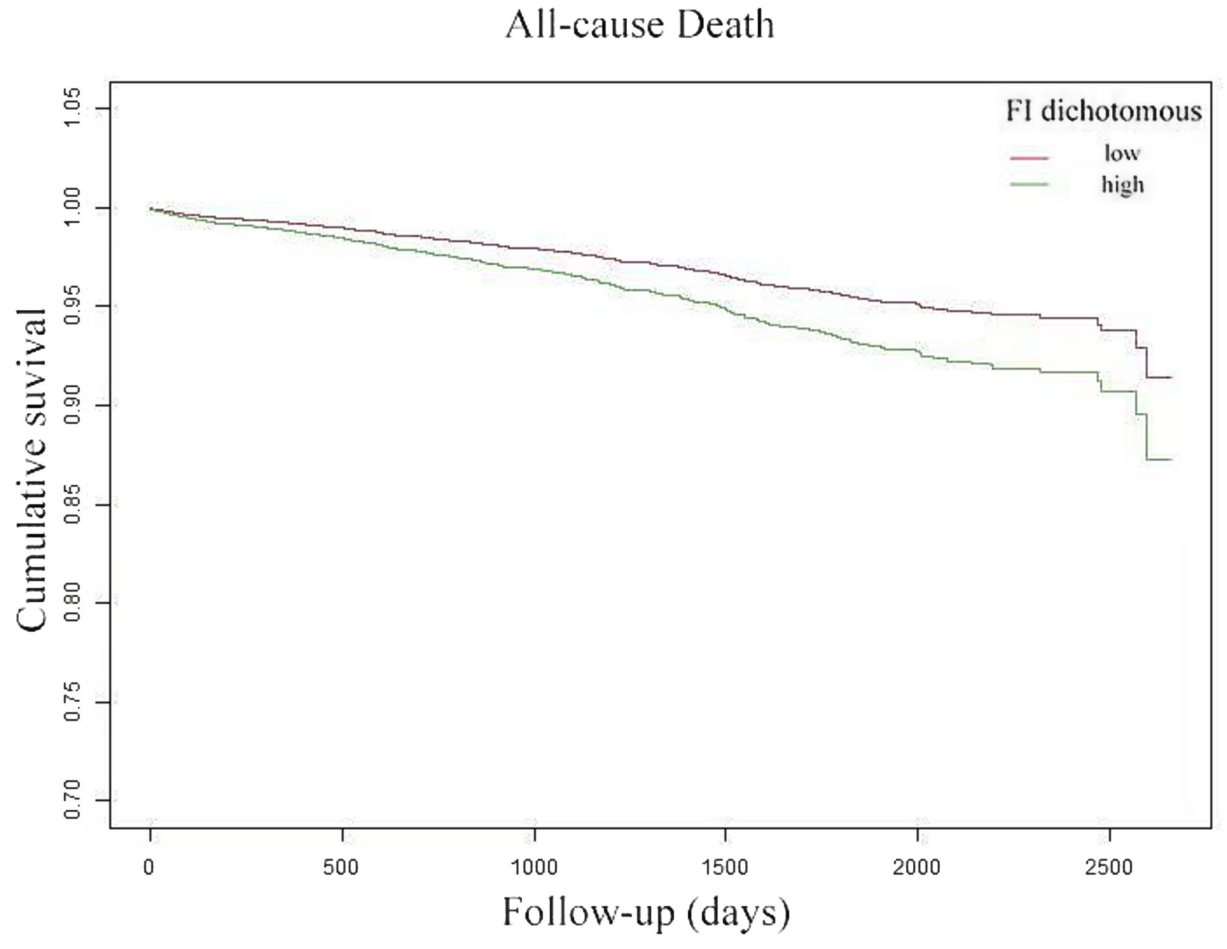
Kaplan–Meier curves reflected the cumulative survival of patients with high FI (green line) and patients with low FI (red line).

The K-M survival curve (Figure 4) demonstrates that patients in the high FI group had a higher ACM rate and lower survival probability than those throughout the observation period. Further, the survival difference between both groups progressively widened over time, suggesting that FI may substantially influence patient survival.

### Subgroup analysis

Subgroup analyses were conducted based on patients’ age, gender, BMI, smoking status, alcohol consumption, ASA score, and fracture types to identify possible confounders in a fully adjusted multivariable Cox regression model. All variables were adjusted in addition to the subgroup variables. Table 4 illustrates that there were no significant stratified interactions (all interaction *p*-values > 0.05), which suggests that the patterns were highly consistent across subgroups.

**Table 4:** Subgroup analysis between FI and ACM.

Subgroup	*N*	ACM^a^ HR (95% CI) p-value	*p*-value for interaction
Age, years, *N* (%)			0.24
Low	2,309	1.10 (1.00, 1.21) 0.06	
High	2,473	1.04 (1.02, 1.06) < 0.01	
Sex, *N* (%)			0.06
Female	3,238	1.03 (1.01, 1.06) 0.01	
Male	1,544	1.08 (1.05, 1.11) < 0.01	
BMI, kg/m^2^, *N* (%)			0.75
Low	2009	1.05 (1.03, 1.08) < 0.01	
High	2014	1.04 (1.01, 1.07) < 0.01	
Smoke, *N* (%)			0.32
No	4,228	1.05 (1.03, 1.07) < 0.01	
Yes	350	1.10 (1.00, 1.22) 0.07	
Drink, *N* (%)			0.06
No	4,341	1.05 (1.03, 1.07) < 0.01	
Yes	237	1.01 (0.81, 1.25) 0.94	
ASA score, *N* (%)			0.38
1	592	1.10 (1.04, 1.17) < 0.01	
2	3,120	1.04 (1.02, 1.07) < 0.01	
> = 3	1,070	1.04 (1.00, 1.10) 0.11	
Fracture category, *N* (%)			0.39
Thoracic vertebra	723	1.02 (0.96, 1.08) 0.69	
Lumbar vertebra	1,290	1.08 (1.03, 1.13) < 0.01	
Wrist	410	1.18 (1.02, 1.37) 0.02	
Proximal humerus	668	0.96 (0.72, 1.28) 0.79	
Thighbone	1,691	1.04 (1.02, 1.07) < 0.01	

a Patients were stratified based on age; sex; BMI; smoke; drink; ASA; fracture category, and additional covariates not included in the stratification were adjusted for in the analysis. HR refers to each 0.01 increase in the frailty index score.

CI, confidence interval; FI, frailty index; ACM, all-cause mortality; HR, hazard ratio; BMI, body mass index; ASA, American Society of Anesthesiologists.

### Sensitivity analysis

**Figure 5:**
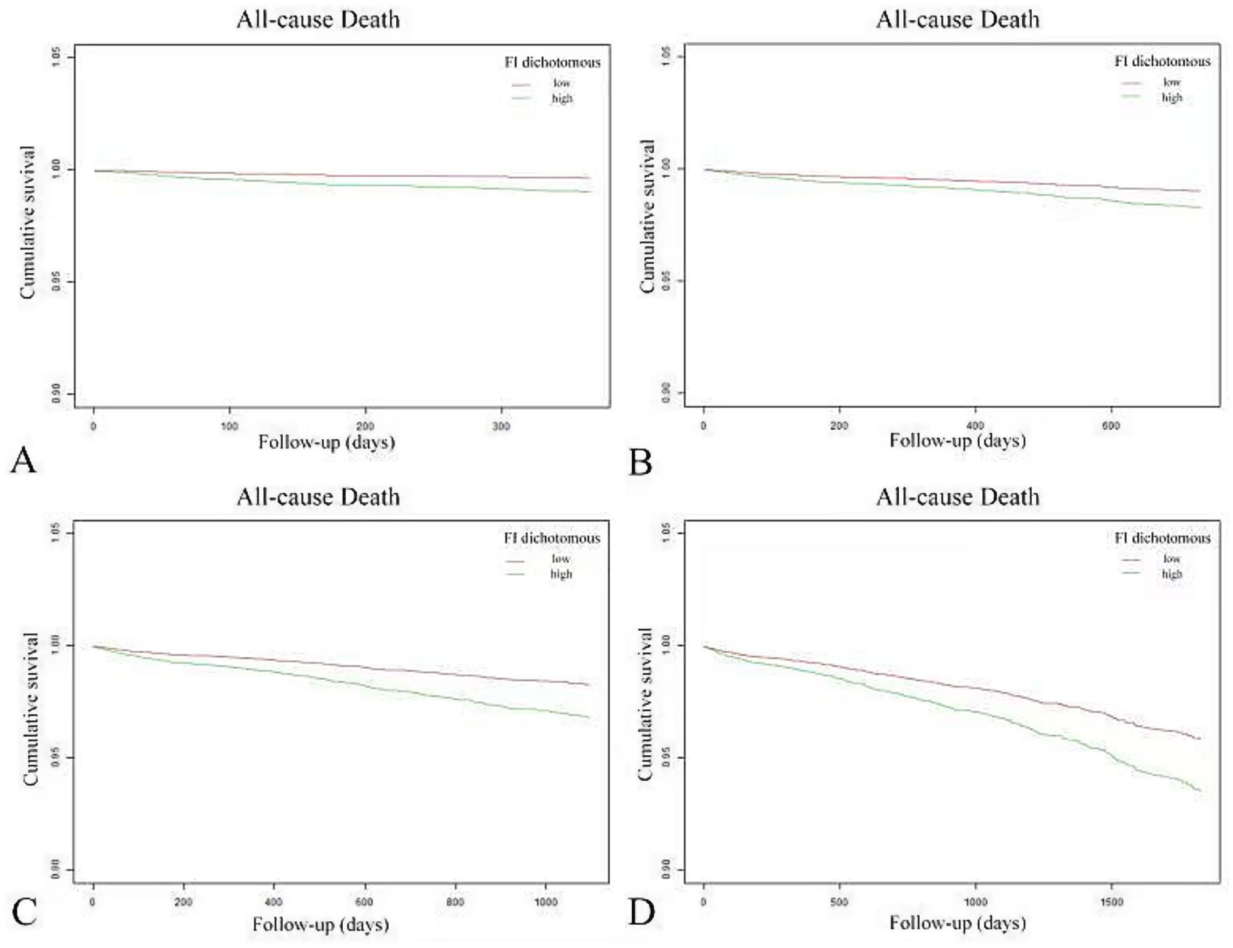
Variable sensitivity analysis of Kaplan–Meier curves for cumulative survival in patients with high FI (green line) and low FI (red line). Panels **(A–D)** correspond to follow-up periods of 1, 2, 3, and 5 years, respectively.

Comprehensive sensitivity analyses were conducted by examining data with varying truncation times. Follow-up data at one, two, three, and 5 years were extracted for regression and survival analyses. Detailed results, presented in Figure 5 and Table 5, confirm the consistency of these findings with the primary study outcomes.

**Table 5:** Sensitivity analysis between FI and ACM.

	*N*	ACM^a^ HR (95% CI)	*p*-value
All-cause mortality			
Censored at 1 year	3,833	1.05 (1.01, 1.09)	0.01
Censored at 2 years	3,833	1.02 (1.00, 1.05)	0.10
Censored at 3 years	3,833	1.02 (1.00, 1.04)	0.18
Censored at 5 years	3,833	1.03 (1.01, 1.05)	<0.01

a The primary analysis using an HR from the multivariable Cox proportional hazards model, with additional adjustment for patient age; sex; BMI; smoke; drink; ASA; fracture category. HR refers to each 0.01 increase in the frailty index score.

CI, confidence interval; FI, frailty index; ACM, all-cause mortality; HR, hazard ratio; BMI, body mass index; ASA, American Society of Anesthesiologists.

## Discussion

This study examined the correlation between the FI and ACM in OPFs. The current findings demonstrated a substantial positive correlation between FI and ACM (HR = 1.05, 95% CI: 1.03–1.07, *p* < 0.01), indicating a 5% increase in mortality risk for every 0.01 rise in FI score. Specifically, each 0.033-point increase in FI score, which approximates 1 additional deficit, was related to a 17% elevation in the risk of ACM (HR = 1.17, 95% CI: 1.10–1.24). These results suggest that higher levels of frailty are related to poorer prognoses in patients with OPFs.

Recent studies have shown a remarkable correlation between frailty and ACM in older patients. For instance, frailty evaluated via the Study of OF (SOF) index was substantially associated with postoperative mortality in older gastric cancer patients undergoing gastrectomy, with frail patients demonstrating over threefold increased risk of death (HR > 3) (35). However, cognitive frailty has been identified as an important predictor of ACM and dementia risk in older adults (HR = 1.93 and HR = 3.66, respectively) (36). Galimberti et al. (32) conducted a multicenter cohort study aimed at developing and validating an FI to predict outcomes in traumatic brain injury patients 6 months post-injury. The study revealed that a higher CENTER-TBI frailty index was considerably correlated with an increased risk of negative outcomes, with the association being more pronounced in patients who were managed in non-intensive care units (32). Collectively, these studies underscore that frailty is not only an independent risk factor faced by older individuals but also substantially impacts clinical outcomes, highlighting the importance of addressing frailty in the management of elderly OPFs.

This study aligns with recent literature, reinforcing the substantial correlation between FI and ACM in older individuals, thereby underscoring the importance of frailty assessment in clinical settings. The present study examines frailty in elderly OPF patients using a comprehensive FI based on 30 health indicators. However, other studies focus on different patient populations, such as those with gastric cancer or traumatic brain injury, and employ different frailty assessment tools, such as the SOF index. Despite variations in study populations and assessment methods, a consistent trend emerges: greater frailty severity is related to an increased mortality risk across diverse patient groups. This reinforces the concept of frailty as a universal independent risk factor that substantially impacts the clinical outcomes of older adults. Therefore, it is crucial to implement targeted screening and intervention strategies to address frailty, thereby enhancing the quality of care and outcomes for vulnerable elderly individuals.

The potential mechanisms underlying the positive association between FI in OPFs and ACM are as follows: First, frailty may decrease the body’s physiological reserve, impairing the patient’s ability to recover from surgical trauma. Second, frailty is related to multi-organ dysfunction, which heightens the risk of complications such as pneumonia, deep vein thrombosis, and cardiovascular events. Finally, frailty is often associated with malnutrition and reduced physical activity, which further exacerbates the progression of OPFs and overall health deterioration, thereby impacting survival rates.

The current findings on the FI offer a valuable tool for identifying frail populations in advance, allowing for developing targeted management strategies for elderly patients with frailty to improve their QoL. These results also underscore the need for further research into accelerated aging in younger populations and the development of relevant screening and intervention programs.

This study has several strengths. It specifically focuses on middle-aged and elderly OPF patients who require inpatient orthopedic surgical treatment. By investigating the feasibility of applying the FI within the field of orthopedics, the study aims to effectively identify high-risk populations, thus providing a scientific foundation for clinical decision-making. Further, key strengths of this study include the use of representative individuals and statistical models that account for multiple substantial confounding factors. However, there are some limitations. For example, this study only focused on middle-aged and elderly patients with osteoporosis who require inpatient surgical treatment, which limits its applicability to young people or OF patients who choose conservative treatment, resulting in a certain degree of selection bias. It is impossible to determine whether the diagnosis of osteoporosis in the patient was made before or after admission. There are still many important factors affecting mortality that were not collected in this study, such as the method of surgical anesthesia, whether the patient’s cause of death was fracture or related complications. These need to be further refined in future research.

## Conclusion

This study revealed that FI and ACM are positively correlated in OPF patients. The results highlight the essential role of frailty assessment in clinical settings, considering that higher FI scores are associated with a higher mortality risk. These findings were consistent with existing literature, affirming frailty as a strong predictor of adverse outcomes in older adults. Future research should aim to elucidate the mechanisms relating frailty to mortality and to develop targeted interventions to improve outcomes for frail elderly patients with OPFs. Overall, the FI represents a valuable tool for identifying high-risk elderly individuals with OPFs, informing personalized treatments designed to enhance survival and QoL.

## Data Availability

The original contributions presented in the study are included in the article/Supplementary material, further inquiries can be directed to the corresponding authors.

## Footnotes

^1^ http://www.R-project.org

^2^ http://www.empowerstats.com
